# Tailoring Transient-Amorphous States: Towards Fast and Power-Efficient Phase-Change Memory and Neuromorphic Computing

**DOI:** 10.1002/adma.201402696

**Published:** 2014-10-09

**Authors:** Tae Hoon Lee, Desmond Loke, Ke-Jie Huang, Wei-Jie Wang, Stephen R Elliott

**Affiliations:** Department of Chemistry, University of CambridgeLensfield Road, Cambridge, CB2 1EW, UK E-mail: sre1@cam.ac.uk; Department of Engineering Product Development, Singapore University of Technology and Design20 Dover Drive, Singapore, 138682; Data Storage Institute, A*STAR5 Engineering Drive 1, Singapore, 117608 E-mail: wang_weijie@dsi.a-star.edu.sg

**Keywords:** phase-change materials, transient-amorphous states, crystallization, semiconductor memories, neuromorphic computing

Phase-change (PC) materials have broad applications in rewritable DVD disks,[1] non-volatile electronic memories,[2] reconfigurable electronics,[3] and more recently, in the area of neuromorphic computing[4–7] by virtue of their accumulative nature of the amorphous-to-crystalline phase transition (ACT) in PC materials, which allows biological-like read/write operations. PC memory (PCM) devices utilize both fast phase transitions between the amorphous (a-) and crystalline (c-) states of PC materials, and accompanying large electrical-resistance contrast, offering feasible intriguing applications in fast/nonvolatile/nanoscale built-in information storage, particularly for their ability to scale down to nanometer length scales.[8–13] However, the overall operation speeds of PCM devices, along with their corresponding power consumption, are intrinsically limited, in particular, owing to the ACT being much slower than the amorphization process. Moreover, much still remains to be done due to the contradictory nature of increasing the ACT speed while at the same time extending the amorphous-state data-retention properties of PC materials.

Current efforts to overcome such limitations are focused on employing material-optimization, or scaling, approaches, such as the development of nanowire-based,[11] or nanostructured,[12,13] PCM devices. However, the improvement of performance with these methods is rapidly becoming impossible due to physical and lithographic constraints. This means that the existing devices based on PC materials will soon reach their ultimate performance limit under the current operating paradigm, insurmountable merely by adopting conventional approaches for improving the performance of PCMs. The need for a precise understanding of the multiple-pulse interaction with PC materials also becomes crucial, especially for neuromorphic-computing applications of PCM technology, such as electronic synapses,[4,5] or bio-inspired arithmetic-computing devices,[6] in which many electric pulses are used in a complicated way to induce phase transitions. However, few systematic researches regarding the physics underlying this interaction have been reported yet.

We report here new methodologies to overcome these limitations by tailoring transient amorphous (TA) states of PC materials. Applying multiple electrical-excitation pulses in a well-programmed manner not only enables the development of a fast, low-power and efficient (parallel-writing) form of PCM, but also provides an opportunity even for achieving biology-like neuromorphic functionalities, which can benefit from a dynamic control of TA-states.

We first demonstrate the temporal evolution of TA-states upon the application of a stimulus pulse. Three different stimulus pulses, each having a different amplitude (*V_St_*) or length (*d_St_*), were employed, while allowing an unbiased time period (*d_TS_*) between the low-voltage/high-voltage (LO-HI) stimulus and ACT pulses. Each stimulus pulse represents a different region in the voltage-width (VW) diagram in **Figure** **1**a, and causes a different influence on the TA states, as shown in Figure 1b. In any case, the TA-phases generated from the stimulus pulse always show shorter minimum ACT lengths (the minimum ACT pulse length for full crystallization) than that for the melt-quenched amorphous (a-) state (60 ns; see Figure S2), which means that exposure to the stimulus pulse always gives rise to faster crystallisation (Figure 1c). In addition, the stronger (and wider) is the stimulus pulse, the shorter the minimum ACT length becomes. A more interesting observation is that, when there exists an unbiased period between two LO-HI pulses, all the TA-states show a spontaneous transition (or relaxation) to other TA-states, each of which presents, progressively, a longer minimum ACT length, up to a characteristic time (∼1 μs).

**Figure 1:**
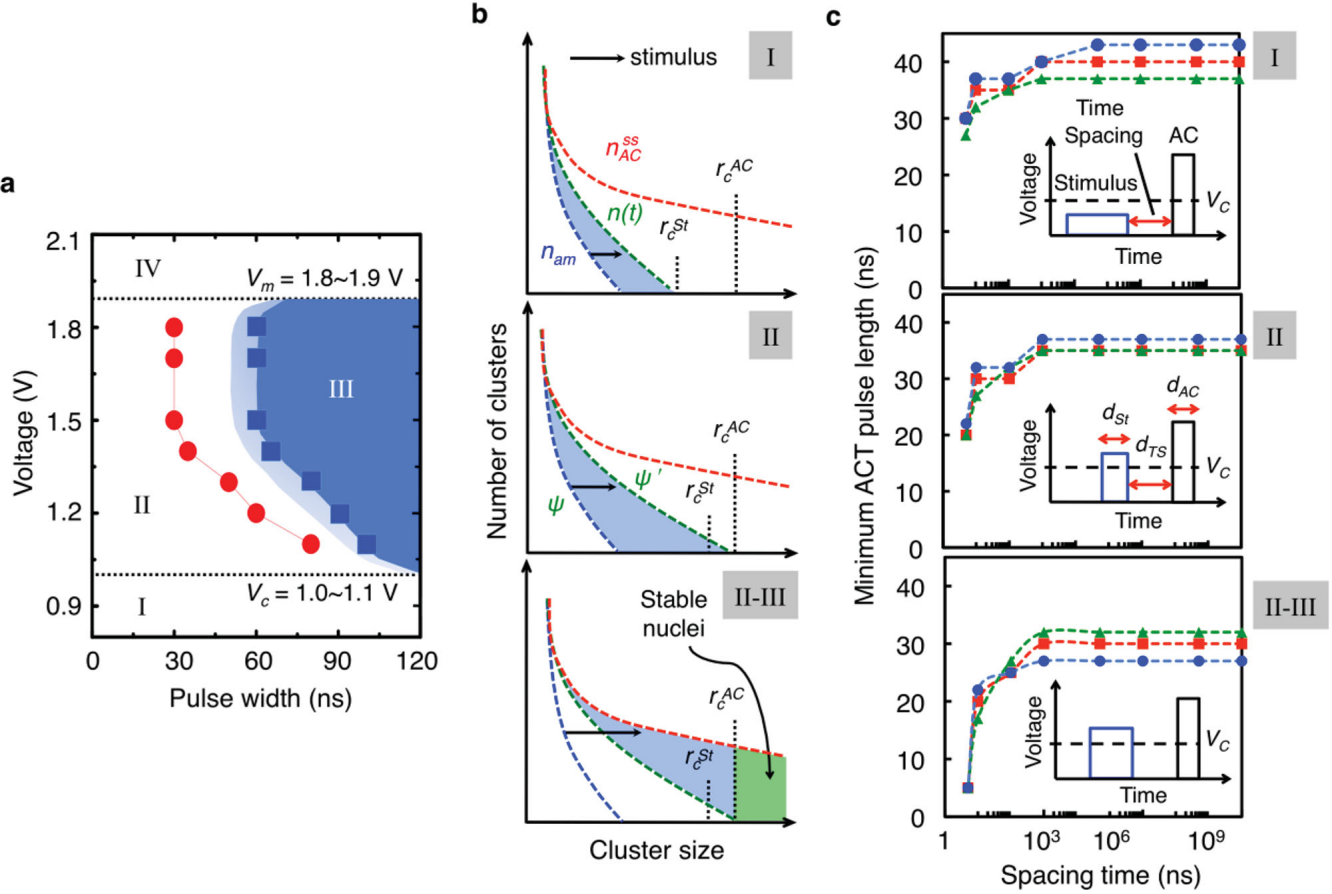
Variation of TA-states upon electrical-pulse stimuli. a) Schematic VW diagram. The VW diagram can be classified into four distinct regions, I-IV (see supplementary information for their characteristics). *V*_m_ and *V*_c_ denote the minimum continuous voltage for melting and full crystallization, respectively. The blue (or red) data points indicate the minimum ACT pulse length before (or 1 sec after) applying a stimulus pulse (1.1 V/90 ns). The light (or dark) blue region represents the pulses that induce partial (or full) crystallization, prior to being exposed to any stimulus pulse. In particular, the boundary region between II and III (i.e., near the partially crystallized zone) is drawn only for a schematic illustration, which is based on the rather coarse data points in Figure S2. b) Schematic description of the evolution of cluster-size distributions upon the application of stimulus pulses corresponding to different regions in the VW diagram. Note the different positions of *r*_c_ at different stimulus (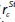) and ACT (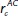) pulses. c) Dependence of the minimum ACT pulse length on pulse parameters for three different test cells. The ACT pulse is denoted in the inset as AC. The stimulus pulses employed in the top, middle, and bottom panels were 0.9 V and 900 ns (region I), 1.1 V and 60 ns (region II), and 1.1 V and 90 ns (region II-III), respectively. The ACT voltage was kept constant at 1.5 V. It is noted that each minimum ACT pulse length for zero spacing time is the same as that for 5 ns of spacing time.

The TA-states can be represented by their cluster-size distribution that form the basis of kinetic theory of nucleation, and the shortening of the ACT time upon a stimulus pulse may be described by their evolution. On the microscopic scale, the TA-states may be described by their degree of medium-range order in the disordered-network structure that fluctuates locally and temporally upon excitations. Numerical computations[14–16] based on the kinetic theory of nucleation have shown that, in response to a temperature change, the cluster population of the melt-quenched amorphous phase *n*_am_ evolves gradually to the steady-state distribution *n*^ss^ at an elevated temperature in a finite amount of time.[17] This time dependence is a consequence of the thermally-activated process during the growth of clusters, which involves atomic attachment or detachment via thermal fluctuations. In PCMs, the cell temperature increases upon the application of electrical pulses via Joule heating. Once a weak stimulus pulse has been applied until time *t′*, the cluster distribution starts to evolve from *n*(*t′*) (Nb. *not* from *n*_am_) upon the application of the subsequent ACT pulse. Accordingly, the expectation time for the clusters to grow beyond the critical nucleation size at the temperature corresponding to the ACT voltage (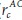) would become shortened when the size of the largest clusters in *n*(*t′*) lies closer to 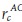 (see Figure 1b). The ACT speed is thus a function of *n*(*t*) at the time of the application of an ACT pulse, which explains the mechanism of faster crystallization for each stimulus pulse, as classified in Figure 1a. The influence of a spatial-temperature distribution, expected to arise in PCM cells upon the application of electrical pulses, may be taken into account in the broad cluster distribution described in Figure 1b, yet the right-most clusters in the distribution (i.e., the largest clusters) mostly determine the crystallization speed upon a subsequent pulse.

The central achievement in this study through the manipulation of TA-states is described in **Figure** **2**, where selected combinations of stimulus and ACT pulses are employed to improve the performance of PCM uniquely. The crystallization time becomes shorter in the presence of the stimulus pulse in proportion to the stimulus pulse length, similar to Figure 1c. Remarkably, however, the overall pulse length, i.e., the sum of the stimulus and the minimum ACT pulse lengths, for full crystallization can be even shorter than that for single-pulse excitation (1.5 V/60 ns, see Figure S2): for instance, crystallization takes just 40 ns overall when stimulus (1.1 V/10 ns) and ACT (1.5 V/30 ns) pulses are applied, as shown in Figure 2a. We estimated the consumed energies for crystallization using single and LO-HI pulse excitations, and they were 76 pJ and 43 pJ, respectively (see Figure S5), corresponding to about a 43% energy reduction along with about a 33% speed improvement. This means that, by applying a weak stimulus pulse immediately prior to a stronger ACT pulse, denoted here as the LO-HI excitation scheme, not only faster but also more power-efficient PCM devices can be developed, compared to using merely the conventional single-pulse operation. Our LO-HI approach is different from the two-pulse excitation proposed by Kang *et al.*,[18] which uses a high-voltage pulse followed by a low-voltage pulse, and whereby only a faster crystallization speed was demonstrated.

**Figure 2:**
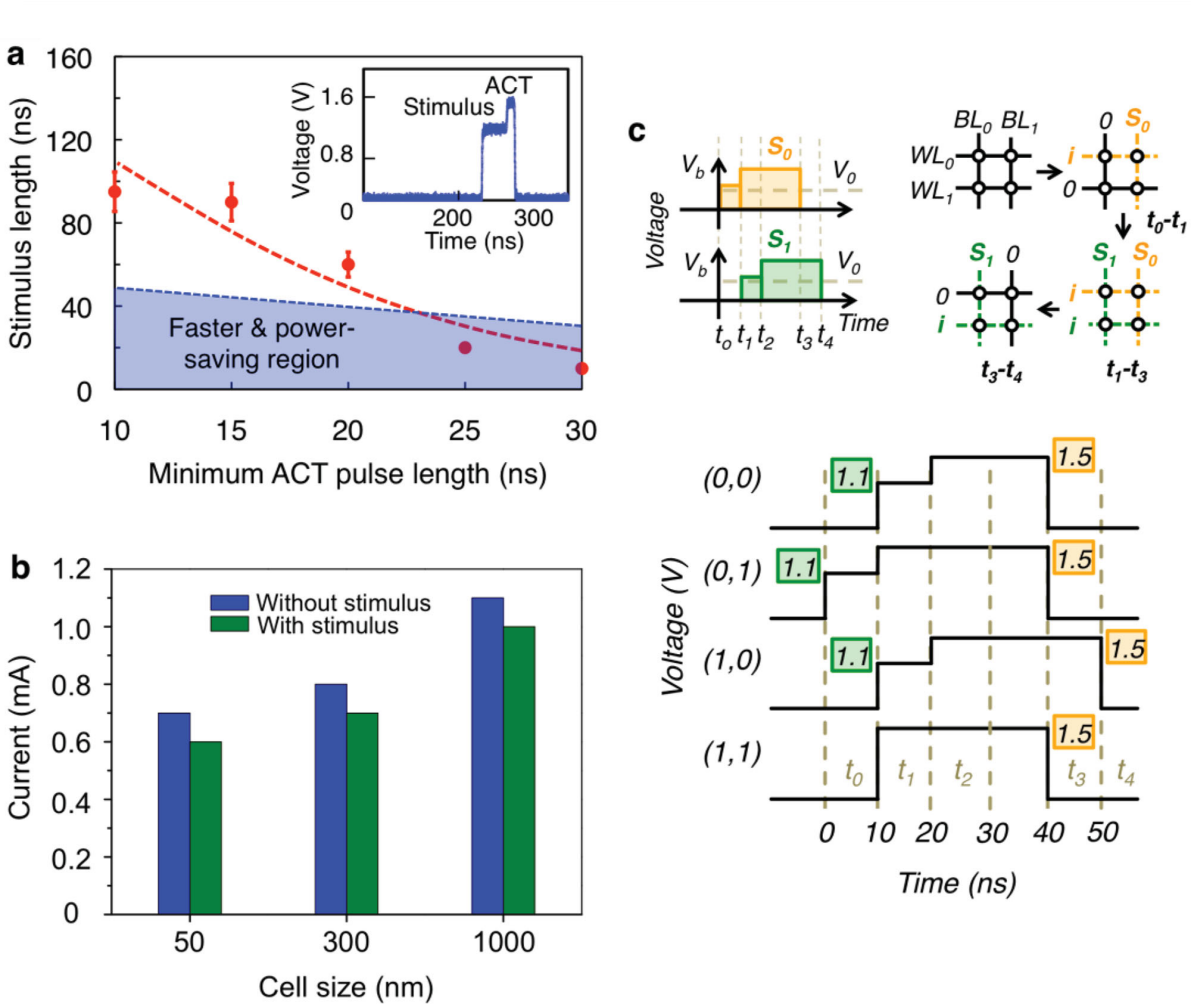
Manipulation of TA-states of PC materials by utilizing electrical-excitation pulses for PCM applications. a) LO-HI pulse excitations for fast and power-efficient SET operations. The inset shows a typical waveform of stimulus and ACT pulses. b) Variation of the minimum current required for full crystallization of cells with different sizes. By applying an electrical pulse (50 ns) with varying voltages, the minimum current required for full crystallization was measured for as-quenched cells without being stimulated and for cells exposed to the stimulus pulse (0.3 V/1 μs) prior to the measurement. c) Parallel-writing scheme in PCM devices. To write a cell, the input *i* is applied to activate all the cells in the row via the WL, while *S*_0_ or *S*_1_ is applied to switch one of the cells in the row from the amorphous to the crystalline state via the BL. *S*_0_ and *S*_1_ are comprised of a stimulus pulse and an ACT pulse.

One may wonder whether the performance improvement of PCM operations via the LO-HI excitation can be achieved even with device miniaturization. To answer this question, we explored scaling properties of the PCM cells. The cells exposed to the stimulus pulse (0.3 V/1 μs) showed the same scaling trend as those that were not exposed (see Figure 2b): the minimum ACT current needed to induce crystallization becomes smaller with cell-size reduction. For each cell size, the current required for crystallization in the simulated cell is smaller than that in the unstimulated cell, which suggests that our two-pulse LO-HI method provides an efficient means to improve the set performance, independently of device miniaturization. This result precisely proves that the manipulation of TA-states provides a completely new way of improving PCM performance beyond the conventional method of device miniaturization. As the LO-HI method is, in principle, feasible for other PC materials, the ultimate SET performance of PCM is anticipated when all these methodologies are appropriately optimized altogether for a given PC material system.

Based on the LO-HI scheme, we were further able to introduce a strategy to achieve parallel writing in memory arrays with the purpose of writing information faster than in the conventional way. With the same principles used in Figure 2a, we selectively switch PCM cells with two pulses (*d_TS_* = 0) by appropriately selecting pulse parameters along word/bit lines. In the example shown in Figure 2c, the PCM cells that we intend to switch (i.e., crystallize) are the (0,1) and (1,0) cells. Here, a specific cell is represented by the first and second numbers in the parentheses, which denote the indices of the word and bit lines, respectively. By selectively activating the cells in different rows along the word lines, applying pulses (10 ns/1.1 V, followed by 30 ns/1.5 V) along the bit lines with time intervals (such as those shown in Figure 2c) results in switching only of the (0,1) and (1,0) cells, while the (0,0) and (1,1) cells remain unchanged, as was intended. This is proved from a wave-form analysis (Figure 2c) of the pulses passing through the cells in the PCM circuit (see Figure S6). In this case, the resultant resistances of (0,0) and (1,1) cells are not affected (i.e., the pulses applied to these cells caused them to remain in region II, as shown in Figure S2), thereby still maintaining a high thermal stability (see Figure S7). The overall time taken for this parallel-writing scheme is 50 ns, much shorter than that in the conventional method (120 ns) that uses a row-by-row operation. This parallel-writing method is especially useful for fast-writing operation, although a trade-off among the speed, power-consumption, and retention properties of PCM cells needs to be considered (see supplementary information).

Although not central to this study, other interesting aspects of the dependence of TA-states on stimulus pulse(s) are now described, besides the PCM application. PCM-emulated synapses[4,5] have shown that the multiple conductance states produced upon the application of electrical pulses are achieved as a consequence of a gradual increase in the crystalline volume in the amorphous matrix, which is analogous to the increase of synaptic strengths in biological synapses.[19] According to Figure 1c, though, when the inter-pulse interval approaches the time scale of the relaxation, the gradual transition of TA-states should be taken into account properly, otherwise unexpected final conductance states would be observed. This may be important, especially for recent studies[4–6] towards ultrafast/power-efficient brain-like computing in PCM cells, where electrical pulses with a shorter inter-pulse interval need to be employed to maximize the operation speed. In addition, although the origin is not well understood at the moment (see supplementary information for detailed discussions), the observed increase, and saturation, of the ACT pulse length with increasing inter-pulse interval in Figure 1c practically resembles characteristic features of biological synapses. The decay of the memory effect with time indicates that the partial crystallization process (or resistance state) seems to be followed by relaxation (see Figure 1). This observation is seemingly analogous to short-term plasticity (STP) characteristics in biological synapses, in which the temporal enhancement in synaptic weight after the pre-synaptic pulse decays quickly. Interestingly, very similar STP characteristics have been observed in Ag_2_S inorganic synapses.[20] Similarly, upon the application of multiple pre-synaptic pulses, the synaptic connection in biological synapses becomes stronger, resulting in long-term potentiation (LTP), which provides an explanation for the mechanism of memory in the brain.[21,22] The results depicted in **Figure** **3** reveal LTP characteristics achieved in PCM cells with multiple-pulse excitations. The resistance (conductance) gradually decreases (increases), as the frequency of pulse trains rises. As biological synapses function via an interaction between two trains of pre- or post-synaptic pulses in diverse frequency regimes,[23,24] our results may provide a ‘biological’ way of achieving LTP. Such a frequency dependence of LTP in PCM synapses has not been observed in other research,[4,5] and this thereby provides a new approach for emulating biological synapses in PCM synapses.

**Figure 3:**
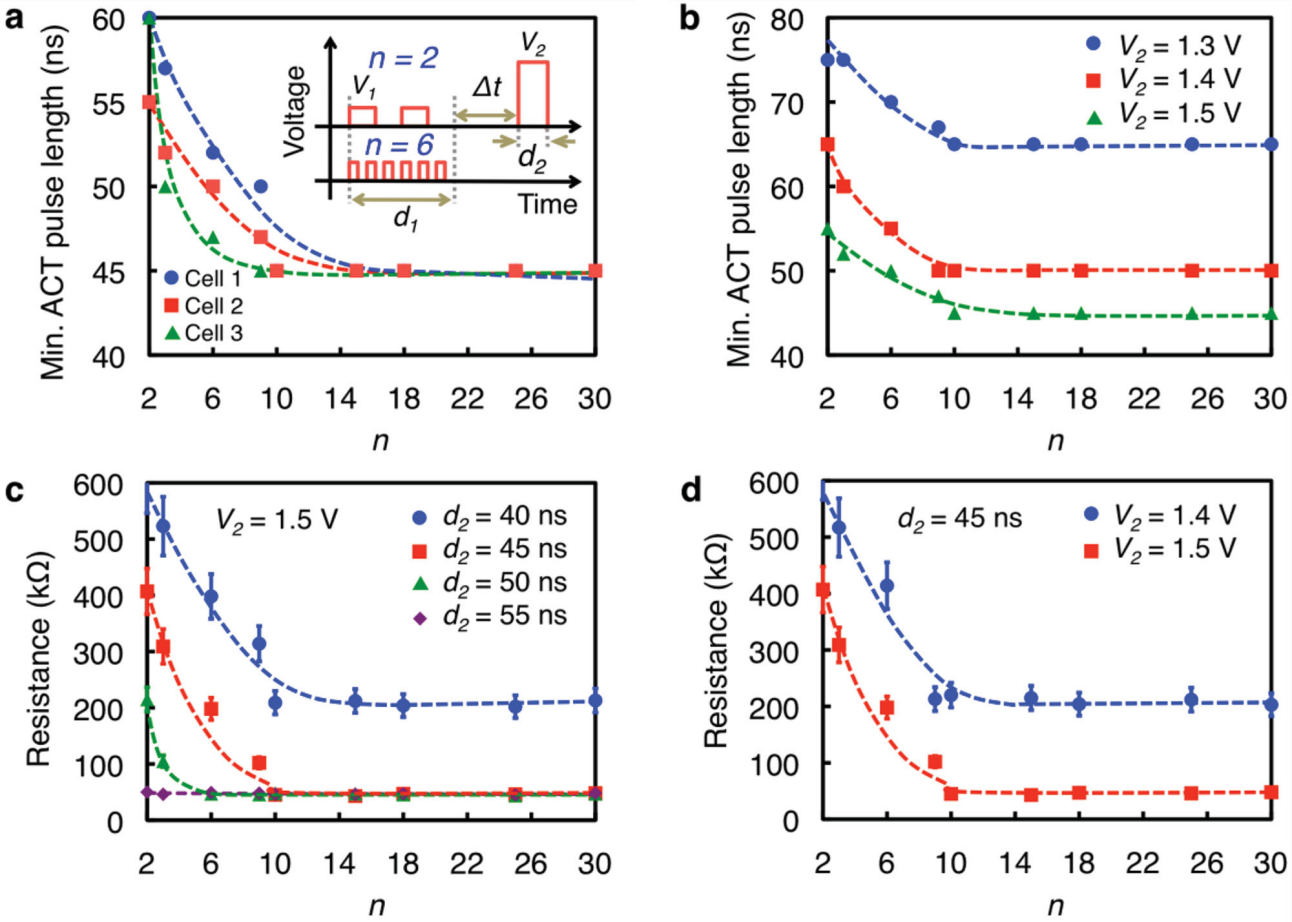
Control of TA-states via frequency modulation of stimulus pulses. Dependence of the minimum ACT pulse length [a) and b)] and cell resistance [c) and d)] on the number of pulse periods *n*. The period number is controlled by varying both stimulus and inter-stimulus pulse lengths within a time frame of 900 ns. The stimulus and inter-stimulus pulse lengths are the same. The number of periods is given by the ratio of the time frame to the sum of the stimulus and inter-stimulus pulse lengths. The stimulus pulse voltages are 0.9 V, and the spacing between the chain of stimulus pulses and the ACT pulse is 1 s. The resistance state of a cell can be modulated by altering three independent parameters (i.e., *n*, *V_2_* and *d_2_*).

Finally, to get an insight into how the LO-HI excitation can give rise to faster, and power-efficient, ACT operation for a certain combination of stimulus and ACT pulses, we consider the temperature dependence of the cluster-growth speed. According to classical nucleation theory,[25] it is anticipated that there may exist a certain range of temperatures at which a maximum cluster-growth speed is observed between *T_m_* and *T_g_* due to the temperature dependence of the atomic diffusivity, as well as of the driving force for the phase transition (∝*ΔT* = *T_m_*-*T*). If this is the case, it is expected on microscopic scales that a dependence of the degree of structural-ordering behaviour on temperature exists. To this end, we performed ab initio molecular-dynamics (AIMD) simulations to study this behaviour.

Indeed, we have found that the degree of structural medium-range order and its evolution, which are closely related to the evolution of the cluster growth, show a dependence on temperature (**Figure** **4**). Three temperatures were carefully chosen to represent material properties with high (400 K), medium (500 K), or low (700 K) viscosities and thermodynamic driving forces, respectively. The diffusion coefficients show as large as two orders of magnitude difference across a temperature range from 400 K to 700 K (Figure 4f) with an approximately Arrhenius temperature dependence, indicating a very sluggish atomic mobility at 400 K. The growth of medium-range order is found to be most significant for annealing at 500 K, the same trend being observed for another set of simulations. Considering both the temperature dependence of diffusivity and the driving force for the phase transition, the small changes in medium-range order at 400 K and 700 K are most likely due to a low atomic diffusivity and a small driving force, respectively, corresponding with the picture of classical nucleation theory. Figure 4e shows an example of the transient crystalline-like cluster that formed during annealing at 500 K, while the atomic configuration at the early stage of annealing (with a lower degree of medium-range order) is shown in Figure 4d.

**Figure 4:**
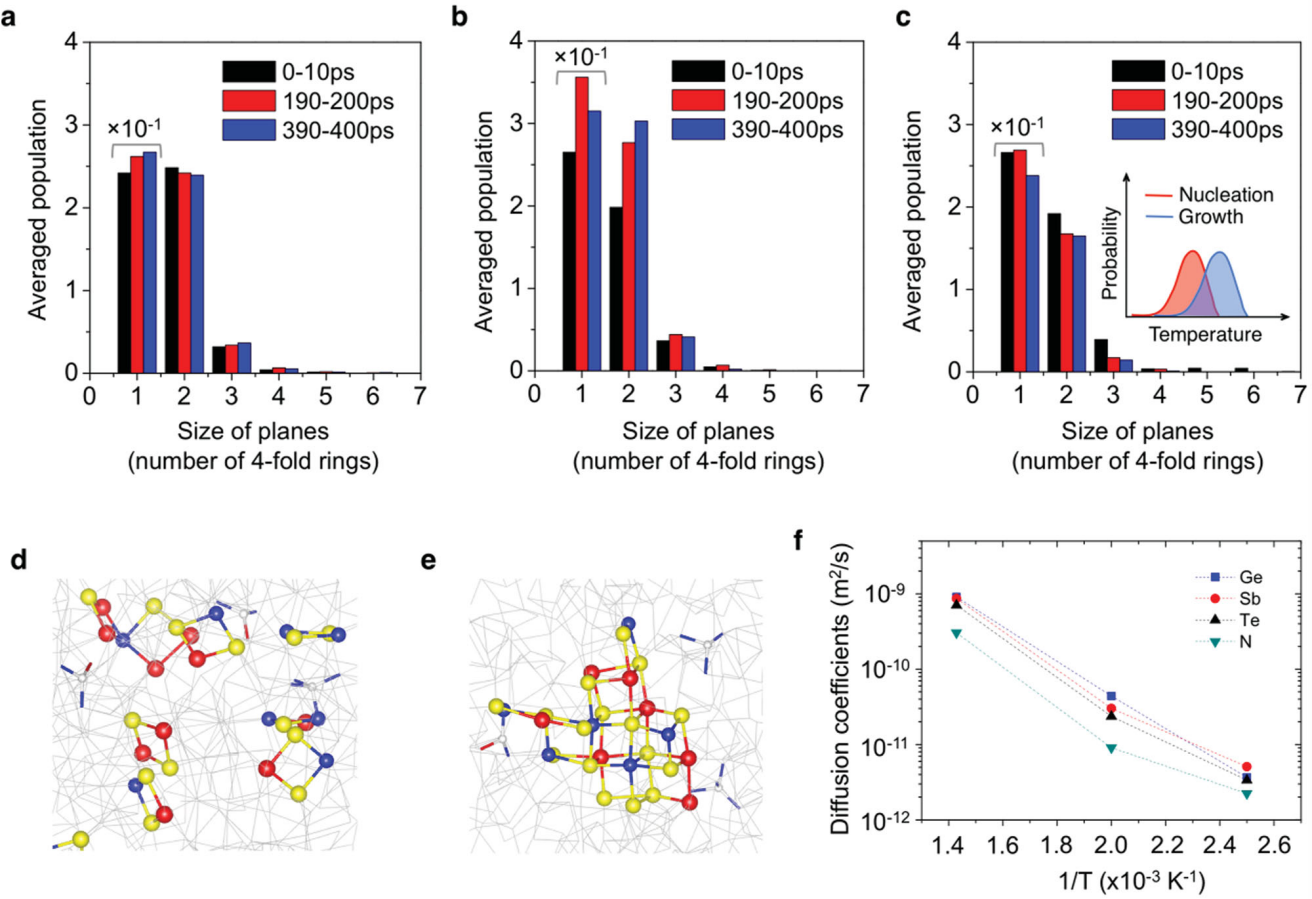
Ab initio molecular-dynamics simulations. Size distributions of 4-fold rings and planes (composed of parallel 4-fold rings) generated in NGST models during different time periods of annealing at: a) 400 K; b) 500 K; and c) 700 K. The number of isolated 4-fold rings is reduced by a factor of 10 for comparison. The number of planes is averaged over the specified time periods. A crystalline-like cluster that formed during annealing at 500 K is shown in e), while the distribution of initial 4-fold rings and planes is shown in d) for comparison. The cluster is composed of Ge (blue), Sb (red), and Te (yellow) atoms with a cubic structure. The probability density of nucleation and growth expected from classical nucleation theory is shown schematically in the inset of c). f) Dependence of diffusion coefficients on annealing temperatures.

These atomic-scale observations support the viewpoint that the speed of cluster growth from *n*_am_ is a function of temperature, as we speculated from the perspective of classical theory. The temperature dependence of the cluster-growth tendency therefore suggests a two-step process of cluster growth in our LO-HI two-pulse excitation scheme, each of the steps showing a different cluster-growth speed that depends on each pulse voltage. Our experimental data clearly show that applying a low-voltage bias of 1.1 V prior to an ACT pulse of 1.5 V reduces the crystallization time, compared to a single-pulse excitation at those voltages. This means that a stimulus pulse of 1.1 V induces a temperature that produces a faster initial cluster growth from the melt-quenched state *ψ* (than does one at 1.5 V), while the later stage of growth from a certain intermediate state *ψ′* becomes faster at the higher temperature corresponding to a pulse of 1.5 V. In the context of classical nucleation theory, we speculate that fast and power-efficient ACT operations may become possible for stimulus and ACT pulses that correspond to the respective near-peak temperatures for nucleation and growth predicted by nucleation theory (Figure 4c, inset). In a consideration of only the speed of crystallization, an extreme case would be the use of a continuous voltage stimulus for region I, which turned out to be an efficient method for decreasing the crystallization times below 1 nanosecond in GST, as we have previously shown.[26] In the current study, we have investigated more generally the impact of multiple pulses on crystallization kinetics of PC materials as functions of the amplitude, width, interval, and the number of sequential pulses. The realization of faster/power-efficient SET operations (*d_TS_* = 0), and of potential synaptic functionalities (*d_TS_* ≠ 0), was only possible from such systematic, intensive investigations over the diverse variables. In this respect, the previous works[18,26] can be considered as special cases of the current generalized approach.

Controlling the dynamic interactions between PC materials and multiple-pulse excitations reported here opens the door to extending the remarkable properties of PC materials towards widely applicable memories and devices. The current description, based on the evolution of TA-states upon the application of diverse pulses in voltage-width space, may provide a framework for developing and modelling devices based on PC materials in a systematic manner. Although we have focused primarily on PCM applications here, the dynamic responses of PC materials to multiple electrical pulses may offer a compelling route towards the development of a new paradigm for ultra-fast neuromorphic computing, especially when combined with future advances in nanofabricated integrated circuits.

## Supporting Information

As a service to our authors and readers, this journal provides supporting information supplied by the authors. Such materials are peer reviewed and may be re-organized for online delivery, but are not copy-edited or typeset. Technical support issues arising from supporting information (other than missing files) should be addressed to the authors.
