# Supplementary material for: Tailoring Transient-Amorphous States: Towards Fast and Power-Efficient Phase-Change Memory and Neuromorphic Computing
Source: Adv Mater. 2014 Oct 9;26(44):7493–8. doi: 10.1002/adma.201402696 (PMC4557048; doi:10.1002/adma.201402696)
Supplement: Supplementary file 1 — Supplementary [file adma0026-7493-sd1.pdf]

Copyright WILEY-VCH Verlag GmbH & Co. KGaA, 69469 Weinheim, Germany, 2014.

ADVANCED MATERIALS

Supporting Information

for *Adv. Mater.,* DOI: 10.1002/adma.201402696

Tailoring Transient-Amorphous States: Towards Fast and Power-Efficient Phase-Change Memory and Neuromorphic Computing

Tae Hoon Lee, Desmond Loke, Ke-Jie Huang, Wei-Jie Wang, and Stephen R. Elliott**

Copyright WILEY-VCH Verlag GmbH & Co. KGaA, 69469 Weinheim, Germany, 2013.

Supporting Information

Tailoring transient-amorphous states: Towards fast and power-efficient phase-change memories and neuromorphic computing

Tae Hoon Lee, Desmond Loke, Ke-Jie Huang, Wei-Jie Wang, and Stephen R. Elliott**

1. Classification of electrical pulses in terms of their effect on the amorphous-to-crystalline phase transition (ACT)

The relationship between electrical pulses and ACT tendency can be represented in the pulse voltage-width (VW) diagram (see Figure 1a). We can classify the electrical pulses into four distinct categories (regions I-IV) in Figure 1a. Each region in the VW diagram has different characteristics. ACT occurs mostly when both the voltage and width of a pulse are such that the voltage is between V_c and V_m and the width is longer than a certain critical value, represented by the (blue) region III in Figure 1a. V_c is defined as the minimum voltage of the continuous stimulus (lasting e.g. hundreds of seconds) that can induce a measurable resistance decrease. V_m is defined as the voltage that can induce melt-quenching of the test cell, eventually resulting in another amorphous phase with a high resistance. Pulses shorter than the critical width (region II) are not able to induce crystallization due to the existence of the incubation period needed for nucleation, although their voltages are higher than V_c. For instance, in our PCM, the stimulus voltage of 1.1 V in Figure 1c corresponds to region II or to region III, where the critical pulse width at 1.1 V is 80-90 ns (see Figure S2b). On the other hand, for voltages lower than V_c (region I), the formation probability of supercritical clusters is too low, due to an insufficient thermal energy resulting from the pulse current, so that most of the clusters are smaller than the critical nucleus size.

The effect of a stimulus pulse on ACT can also be synthetically presented in the pulse voltage-width (VW) diagram (Figure 1a). The observed shortening in the minimum ACT pulse length, then, can be represented by a shift of the critical pulse width toward a shorter value, which means that an ACT pulse (red circles in Figure 1a) shorter than that for the unbiased case (blue squares in Figure 1a) can now induce crystallisation after the application

of a stimulus. Regions I and II, here denoted as an incubation-pulse zone, are of great interest as the physical properties of amorphous phases, notably the electrical resistance, are not significantly affected by the application of stimulus pulses, while the resulting microscopic structural changes greatly reduce the ACT time upon the application of an accompanying ACT pulse. Stimulus voltages corresponding to region III or the boundary II-III, denoted as a crystallisation zone, enhance the ACT speed most effectively upon the application of an ACT pulse.

Each region in the VW diagram can also be characteristically represented in terms of cluster-size distributions: (i) the temperature increase required for crystallization is not high enough with the pulses corresponding to region I, such that the largest clusters in $n(t)$ lie below the critical size (r_c), and nucleation events hardly occur with these pulses; (ii) region II represents the pulses whose resulting steady-state cluster distribution has a large population of clusters exceeding r_c but, because of short pulse widths, the cluster growth is limited and most clusters are still smaller than r_c; and (iii) measurable crystallization occurs only for pulses in region III which have sufficient amplitudes and widths to generate a substantial population of large supercritical clusters. The cluster distribution in glasses during annealing at high temperatures has been reported before [S1-S3]. The cluster-distribution lines in Figure 1b are schematically drawn, based on these theoretical considerations as well as experimental observations.

Due to an uncertainty in the critical-nucleus size (and the surface energy), we have attempted to describe the critical-nucleus size only qualitatively in Figure 1b. Assuming that the dependence of the surface energy both on temperature and cluster size is relatively small, the relative size of the critical nuclei (r_{c1} and r_{c2}) at different temperatures (T_1 and T_2) can be quantitatively estimated within the framework of classical nucleation theory as $r_{c1}/r_{c2} \sim (T_m\text{-}T_2)/(T_m\text{-}T_1)$. Since the stimulus pulse at a low voltage induces a lower temperature (T_1) than a set pulse (T_2), the corresponding critical nucleus size for a stimulus pulse is always smaller than that for a set pulse by a factor of $(T_m\text{-}T_2)/(T_m\text{-}T_1)$.

2. Electrical Characterization

a) Device fabrication and structure

Test cells were fabricated using conventional lithography. Each patterning step was accomplished using a 365 nm photolithography system (Cannon Stepper FPA-2000 i1), followed by material-deposition and lift-off processes. All of the materials were deposited

using composite targets in a DC magnetron sputtering system (Balzers Cube). Nitrogen-doped $Ge_2Sb_2Te_5$ (NGST) films were deposited by sputtering from a composite GST target and concurrently in flowing nitrogen gas at a constant N_2/Ar flow rate of 0.2. The NGST films were characterized using X-ray photoelectron spectroscopy (XPS), which showed that the nitrogen concentration in the films is around 3.5 at. %. In the memory cell, a SiO_2-on-Si substrate was used as the starting structure, on which a 200 nm thick TiW bottom electrode was deposited and patterned. An insulating layer, comprising a 30 nm thick layer of SiO_2 was deposited, and etched to form vias with diameters of 1 μm. 30 nm thick NGST was filled into the vias to form the active PCM regions. Finally, a 200 nm thick TiW top electrode was deposited to complete the structure.

Figure S1a shows the cell structure of a sample in the lateral/cross-sectional plane, whereby the cell is deposited on a SiO_2-on-Si substrate. The cell has a pore-like structure that is comprised of a 30 nm thick phase-change element (NGST), which is sandwiched by the top and bottom electrodes (TiW) with thicknesses of 200 nm. The phase-change element is confined in a 1-μm-wide via formed by an insulating layer (SiO_2) with a thickness of 30 nm. The electrodes are used to connect the cell to the external circuitry for the electrical measurements. The insulating layer is used to provide electrical/thermal insulation for the cell.

b) Electrical measurements

The electrical performance of the cell was investigated using a conventional testing system [S8] that is comprised mainly of a nanosecond-pulse generator (Tektronix), a digital oscilloscope (Agilent Technologies), and a probe station, as shown in Fig. S1b. The nanosecond-pulse generator has the specifications of pulse durations ranging from 5 ns to 900 ns, a rise time of less than 3 ns, and a maximum amplitude of 5 V. The PCM is connected to the generator/oscilloscope via low-capacitance cables (~0.2-3 pF) and a series resistor, $R_l = 50$ Ω. The voltage V_b is probed at the node B at the bottom of the cell, which allows for extraction of the current in the load resistor, given by $I = V_b/R_l$. The upper limit of the time constant of the RC circuit is estimated to be ~several 10s of ps. The full-width, half-maximum time duration (FWHM) of the pulse (see Figure S5, conventional one pulse, and step pulse) was measured at V_a (see Figure S1b), and this was used to characterize the speed of the PCM switching. The waveform of the pulse obtained at V_a also reflects the exact voltage pulse that is applied to the PCM, taking into account the capacitance/inductance of the probe/circuitry/connectors. Figure S1c further shows the waveforms of the pulse signal

measured before (V_a) and after (V_b) the PCM. From the measurement results, we can see that the FWHMs of the waveforms measured at V_a and V_b are almost the same. As the signal measured at V_b has passed through the PCM, this confirms that the duration of the pulse experienced by the PCM is almost identical to that of the pulse entering the PCM. A comparison of the shapes of the pulses measured at V_a and V_b also confirms that the electrical/parasitic-capacitance effects in the circuit/PCM are small/negligible, and in the case where they exist near the end of the pulses, they dissipate in less than 10 ns, consistent with the times reported by other groups [S7,S9,S10].

c) Long/short-term stability and endurance characteristics

We studied the stability of the reset (amorphous) state over an extended period of time (see Figure S7a). The cells were annealed at temperatures varying from 443 K to 473 K, and the ACT times were recorded for the normal and stimulated cells. From a data extrapolation, the stimulated cells are estimated to withstand ACT for up to 10 years at 362 K, which is similar to that of normal unbiased cells (i.e. up to 10 years at 368 K). In Figure S7b, we show the evolution of resistance for normal and stimulated cells in the reset (amorphous) state with time. In the experiment, a stimulus pulse of 1.1 V and 60 ns was applied to the stimulated cell. Both normal and stimulated cells show an increase in resistance ('drift') with time. The resistance-drift power-law coefficient, *v,* used as a measure of the degree of increase in resistance, is given by:

$$R = R_0(\frac{t}{t_0})^v,$$

where *R* and *t* are the resistance and time, respectively, t_0 is an arbitrarily chosen zero time, and R_0 is the resistance at t_0. Both cells show similar resistance-drift coefficients, which is consistent with the properties of good PC materials that can efficiently relax mechanical stress [S11, S12]. Figure S7c illustrates the endurance of a test cell that was switched using 5 ns ACT and reset pulses, preceded by 1 μs stimulus pulses. The cell maintained reversible and stable switching for 2×10^8 cycles. The cells were switched between the low- and high-resistance levels of 10 kΩ and 200 kΩ, respectively.

3. Possible explanations for the increase of the minimum ACT pulse length with increasing the inter-pulse interval

a) Electrical/thermal effects

We now discuss thermal effects in PCM devices (see above for a discussion of electrical effects). We have used one of the conventional cell structures, as employed by many other research groups [S13,S14]. The pulse width needed to switch the cells without applying the stimulus using our current cell structure was found to be about several 10 ns (depending on the voltage applied), which is about the same as compared to the set speed achieved with the other cell structures [S15,S16]. This means that the effect of heat retention in our cells is similar to other cell structures. The thermal time constant of a PCM is difficult to measure experimentally due to the fast heating/cooling times of the PCM material/structure. It has been estimated mainly using two-dimensional electro-thermal device simulations, although the parameters tend to vary by multiplicative constants [S17]. The thermal time constant of the PCM cells has been estimated to be a few ns [S15,S16], and since our PCM cells have a similar heat-retention property as these cells, an estimate for the thermal time constant for our cells may be inferred to be also within the same range.

We further discuss the possibility that the device crystallizes more easily because it retains more heat for smaller pulse separations. For this picture to be plausible in explaining our experimental results, one expects a certain constant increase in the crystallization time comparable to the thermal time constant. For instance, let us consider the case in which the minimum ACT pulse length is measured as a function of the spacing time after the application of a stimulus pulse of 1.1 V with a 60 ns width (corresponding to the middle panel in Figure 1c). Comparing the minimum ACT pulse length for d_{TS} = 5 ns with that for d_{TS} = 1 μs, a 15 ns of increase in the minimum ACT pulse length is observed with the spacing time. If one expects that this is due to a thermal effect, then 15 ns would be closely associated with the thermal time constant of the device. If we compare this result with the case of a stimulus pulse of 1.1 V with a 90 ns width (the bottom panel in Figure 1c), which is slightly longer than the above pulse, the increase in the minimum ACT pulse length is found to be 25 ns. If this were to be solely due to the thermal effect, one would expect the *same* increase in the minimum ACT pulse length for both cases, which is contrary to our observations (Figure S4). Moreover, an estimation of the thermal time constant to be ~ns seems reasonable if one considers that only a 10 ns duration of the stimulus pulse at 1.1 V with d_{TS} = 0 resulted in a huge improvement in the energy efficiency and set speed during the following ACT pulse, as shown in Figure 2a. Otherwise, there would be little change. These results indicate that the main origin of the increase of the minimum ACT pulse length seems to be not due to electrical/thermal effects, but due to an intrinsic material property.

b) Interface-shrinkage model

The origin of the increase (or relaxation) in the ACT time with d_{TS} is not clearly understood in the framework of classical nucleation theory. A spontaneous relaxation is anticipated in this theory only when n^{ss} at room temperature would lie much below $n(t)$ [and the critical size of nuclei, r_c, would lie above the size of the largest clusters in $n(t)$], such that large clusters formed upon the application of stimulus pulses could be resolved in the amorphous matrix during the unbiased period, leading to a shift of $n(t)$ to the left in the cluster-distribution diagram. Considering both the expected temperature dependence of r_c ($\propto 1/\Delta T$, where ΔT is the degree of undercooling) and of n^{ss} (the largest size of clusters in n^{ss} is expected to be much larger than r_c), it seems that neither r_c nor n^{ss} at room temperature may satisfy these conditions. A quantitative comparison between the rate-equation approach and experimental observations is somewhat questionable due to the uncertainty in the input parameters for the calculation (such as interfacial energy, viscosity, etc.) that are presumably functions of the cluster size, and/or of the temperature. The uncertainty should worsen in the case of a complicated (and not precisely known) thermal history in the material, and of the preferential nitride formation and its segregation at grain boundaries for NGST [S5, S6]. Therefore, we treated here the crystalline-cluster distributions qualitatively within the framework of classical nucleation theory.

A non-classical interpretation that we can consider could be an interface-shrinkage model in which the evolution of glass-cluster interface as a function of temperature is involved. (Such an influence of interfaces is largely overlooked in the current nucleation theory.) The necessity of taking into account the interface has been invoked in recent nucleation theories [S18,S19]. Although their applicability to explain the current observation is still unknown, a stronger deviation from the classical theory is predicted with more supercooling. An interesting observation as to this interface has been gained from recent *ab initio* molecular-dynamics simulations of GST [S4], where the growth of the interface region, with a planar (crystalline-like) symmetry, facilitated faster crystallization during annealing, even without any growth of clusters having a cubic symmetry characteristic of the metastable rocksalt structure. This result emphasizes the possible role of interfaces in cluster-growth dynamics at lower temperatures and nucleation in GST. The inverse transition with a temperature decrease, in this context, would be the shrinkage (and disordering of the short-range order, as inferred in Ref. S4) of the interfacial region, which is, in principle, in line with the increase of the ACT pulse length with the unbiased period. If we denote the TA-state

existing directly after the stimulus pulse as ψ', the decay can be described by a gradual structural relaxation of the state ψ' during the pulse-separation time to the low-temperature TA-state ψ'', whose microscopic structure is less suitable for fast ACT than that of the state ψ'.

Because of current limitations in experiment, simulations, and theoretical understanding, however, the precise origin of this behaviour is not known. In this respect, the interface-shrinkage model is still hypothetical and remains as an open question that needs to be addressed in follow-up studies.

4. Advanced Writing Scheme

a) Stimulating circuit

In Figure S6a, we show a schematic diagram of a stimulating circuit for the test cells. The cell is connected to the word line (WL) and the bit line (BL). The bit line is further connected to the stimulus voltage (V_B) and write voltage (V_W). To stimulate the cell, the signals from the WL and BEN (bias-enable) are first activated to form a conductive path for the stimulus pulse (V_B) to pass through the cell. To set the cell, the WL and WEN (write-enable) are activated to form a conductive path for the signals from the BL to pass through the cell.

b) Characteristics of parallel-writing scheme

We discuss the use of the LO-HI method in the pipelined (PL) write scheme to achieve fast writing of PCM cells in a memory array. We find that the PL-write scheme has energy-loss but little/no scalability issues. Figure S6b shows the write waveforms employed in the PL-write scheme where, in a worst-case scenario for a large array, the cells in four rows and four columns are turned on simultaneously. Among these sixteen cells, at least three of them would be written, and one would be under a bias. The energy loss for the array can be accounted via the currents that flow/leak via the other twelve cells in the block. The rest of the cells in the array would be unaffected. Assuming that the same PL-write scheme is used in a larger array, the number of affected cells would remain unchanged, regardless of the size of the array, meaning that such a write scheme would have little/no scalability issues. As such, the timing of the signals for the PL-write scheme can be controlled via using conventional peripheral/accompanying devices. In the same worst-case scenario, four row addresses and four column addresses are enabled each time, and they are enabled at each different cycle.

The registers may be used to latch the addresses of the decoder output for the four cycles. The decoders can be the same as those used in the conventional row-by-row (RR) write scheme. In addition, the random-access ability of the PL-write scheme would be identical to that for the conventional RR-write scheme, since the address decoders employed are the same. Furthermore, the voltage levels can be controlled via a memory controller, although it will be more complicated since more voltage levels are required.

We further note that there is a trade-off between the speed, and power and retention properties of the PCM cells in an array. During the writing of a block of cells in the array, for instance one of 2×2 cells, as shown in Figure 2c, two cells positioned at the coordinates ‘01’ and ‘10’ can be written in 50 ns using the PL-write scheme, which is about two and half times shorter than by using the conventional one-pulse RR-write scheme (120 ns) (see Figure S6b). However, the power consumption for such a PL-write scheme is higher than that for the conventional RR-write scheme. In the same block of PCM cells, the energy consumed/loss to write a cell in the block can be estimated via a simple calculation of the area under the subjected-signal/pulse waveforms. In the conventional RR-write scheme, the energy-consumed value for writing a cell in the block would be $9t_p$ ($1.5 \times 6t_p$), where t_p is defined as the time period of 10 ns. For the PL-write scheme, the energy-consumed value for writing a cell is given by $5.6t_p$ ($1.5 \times 3t_p + 1.1 \times t_p$), while in a worst-case scenario, the energy-loss values for the other three ‘unselected’ cells in the block would be $16.8t_p$ ($3 \times 1.5 \times 3t_p + 3 \times 1.1 \times 3t_p$)]. The total energy consumed/loss value for the PL-write scheme is thus given by $22.4t_p$ ($5.6t_p + 16.8t_p$), which is about two and half times higher than that for the conventional RR-write scheme. In addition, via the studies/calculations earlier, although the cells in a block can be written in a shorter time for the PL-write scheme compared to that for the conventional RR-write scheme, due to the accumulative-transition nature of the PCM material, the unselected cells would be more easily disturbed/switched by the bias/write signals after subsequent write cycles in the former write scheme than that in the latter write scheme.

To reduce/eliminate these energy-loss/write-disturb issues, a low-power modified-RR-write scheme may be employed to write the PCM cells in a memory array. In this modified RR-write scheme, a cell is written using a two-step pulse in a row-by-row manner. To write a cell in a row of the array, for instance, while the word line WL is turned on, the bit lines BEN followed by WEN are enabled to bias and write the cell, respectively. The unselected cells in the same row, as well those in other rows, would not be written, meaning that little/no energy loss will be incurred. In addition, since the unselected cells would remain unchanged, regardless of the array size, such a write scheme would have little/no scalability issues. The

energy-consumed/loss to write a cell in the modified RR-write scheme is also lower ($5.6t_p$) than that in the conventional RR-write scheme ($9t_p$). The same is also true for the time required to write the cell using the former write scheme (40 ns) compared to that for the latter scheme (60 ns). It should be noted that, however, to write a block of cells in the array, for example, the same cells (01/10) in the earlier 2×2 matrix, the time needed to write these cells would be longer for the modified RR-write scheme (80 ns) compared to that for the PL-write scheme (50 ns).

Finally, in the modified-RR write scheme, the timing of the signals can be controlled such that one row address and multiple column addresses are enabled each time. The registers can be used to latch the addresses of the decoder output for one cycle. The decoders are the same as those used in the conventional RR-write scheme. The random-access ability of such a write scheme would be identical to that of the conventional one, since the address decoders used are the same. The voltage levels can be controlled via a memory controller, although it will be more complicated than the conventional RR-write scheme as more voltage levels are required.

Overall, these studies suggest further that the LO-HI scheme can be exploited to achieve reconfigurable information storage, whereby varying write schemes can be employed using a single PCM array for high-speed memory applications.

c) Conventional writing method

In the conventional PCM method, the storage of information is done sequentially, as shown in Figure S6c. Information can be stored, for instance, in the cells (0,1) and (1,0) of a 2×2 array of cells, where the first and second numbers in the parentheses denote the indices of the word line (WL) and bit line (BL), respectively. The signals S_2 and S_3 are applied to switch the cells (0,1) and (1,0), one after another, from times t_0 to t_5 and from t_5 to t_6, respectively. Initially, all the cells are in the amorphous state. First, to write cell (0,1), the input i is applied to activate all the cells in the first row via WL_0, while the signal S_2 is applied to the cell in the second column via BL_1, which causes the cell to switch from the amorphous to crystalline state. Subsequently, to write cell (1,0), the input i is applied to activate all the cells in the second row via WL_1, while the signal S_3 is applied to the cell in the first column via BL_0, which causes the cell to switch from the amorphous to the crystalline state. Both S_2 and S_3 employ long pulses to switch the cells, which are typical of those found in region III of the pulse voltage-width (VW) diagram in Figure 1a.

d) Modified conventional method

It should be noted that the conventional method can be modified to write cell (1,0) while cell (0,1) is being written. Although the overall writing time can be reduced to 80 ns (starting time difference of 20 ns), the total overlap time (40 ns), which is defined as the duration of the pulses delivered to the untargeted cells, would be larger than that for the new method (30 ns), which means that the delivered pulse duration would be closer to region III, which would reduce the amorphous-phase stability of the untargeted cells (see ref. S20).

5. *Ab initio* molecular-dynamics simulations

Simulations were performed using the Vienna Ab initio Simulation Package (VASP) [S21]. The projector augmented-wave (PAW) method [S22] with the Perdew-Burke-Ernzerhof (PBE) exchange-correlation functional [S23] was used. A 183-atom model of NGST, including three nitrogen atoms, was simulated in cubic supercells with periodic boundary conditions. The plane-wave energy cutoff was 325 eV. All the outer s and p electrons were treated as valence electrons. The time step for molecular dynamics was 3 or 4 fs, and the temperature was controlled by a Nose thermostat algorithm. The density used was 6.11 g/cm^3. Initial random atomic configurations were mixed at 3000 K, maintained at 1200 K for tens of ps, and then quenched to generate amorphous configurations with a quench rate of -15 K/ps. The amorphous models were then annealed at 400 K, 500 K and 700 K. The definition of structural units, such as 4-fold rings and planes of such rings, can be found elsewhere [S4].

References

S1. K. F. Kelton, A. L. Greer, Nucleation in condensed matter: Applications in Materials and Biology (Elsevier, Oxford, 2010).

S2. B. –S. Lee *et al.*, *Science* **2009**, 326, 980.

S3. K. Darmawikarta *et al.*, *J. Appl. Phys.* **2012**, 112, 124907.

S4. T. H. Lee, S. R. Elliott, *Phys. Rev. Lett.* **2011**, 107, 145702.

S5. R. Kojima *et al.*, *Jpn. J. Appl. Phys.* **1998**, 37, 2098.

S6. T. H. Jeoung, M. R. Kim, H. Seo, J. W. Park, C. Yeon, *Jpn. J. Appl. Phys.* **2000**, 39, 2775.

S7. D. Ielmini, D. Mantegazza, A. L. Lacaita, A. Pirovano, F. Pellizzer, *IEEE Electr. Dev. Lett.* **2005**, 26, 799.

S8. L. P. Shi *et al.*, *NVMTS*, 2005, pp. 115-120.

S9. M. P. Shaw, I. J. Gastman, *Appl. Phys. Lett.* **1971**, 19, 243.

S10. D. Adler, M. S. Shur, M. Silver, S. R. Ovshinsky, *J. Appl. Phys.* **1980**, 51, 3289.

S11. A. Pirovano *et al.*, *IEEE Trans. Electron Devices* **2004**, 51, 714.

S12. I. V. Karpov *et al.*, *J. Appl. Phys.* **2007**, 102, 124503.

S13. M. Breitwisch *et al.*, *Symp. VLSI Tech.*, **2007**, 100.

S14. D. H. Im *et al.*, *IEDM Tech. Dig.* **2008**, 1.

S15. S. Lai, *IEDM Tech. Dig.* **2003**, 10.1.1.

S16. G. Atwood, R. Bez, *DRC Tech. Dig.* **2005**, 29.

S17. J. Reifenberg, E. Pop, A. Gibby, S. Wong, K. Goodson, *ITHERM* **2006**, 106.

S18. L. Gránásy *J. Non-Cryst. Solids* **1993**, 162, 301.

S19. C. K. Bagdassarian, D. W. Oxtoby, *J. Chem. Phys.* **1994**, 100, 2139-.

S20. S. Braga, A. Cabrini, G. Torelli, *IEEE Trans. Elect. Dev.* **2011**, 58, 517.

S21. G. Kresse, J. Hafner, *Phys. Rev. B* **1993**, 47, 558.

S22. P. E. Blöchl, *Phys. Rev. B* **1994**, 50, 17953.

S23. J. P. Perdew, K. Burke, M. Ernzerhof, *Phys. Rev. Lett.* **1996**, 77, 3865.

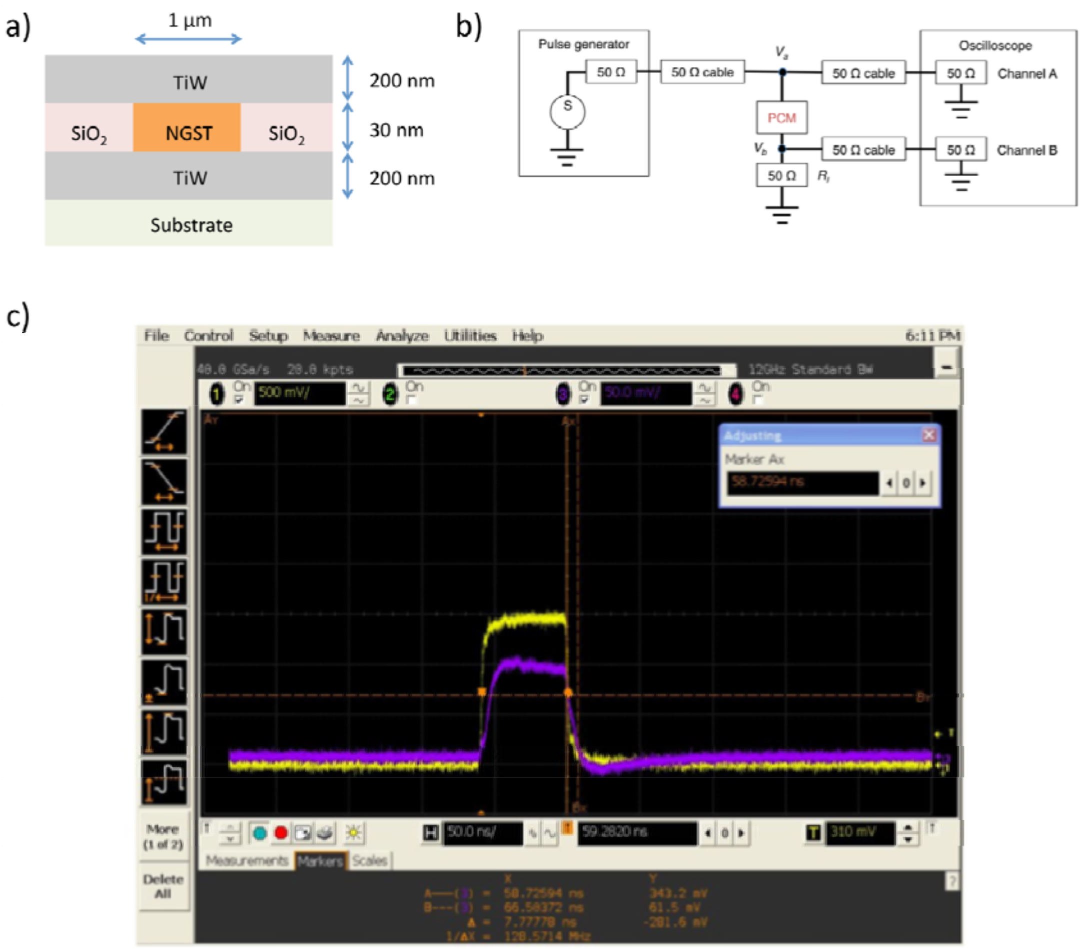

Figure S1. Structure and electrical measurements of PCM cells. (a) Structure of a cell in the lateral/cross-sectional plane. (b) Schematics of the electrical measurement setup, including a pulse generator, PCM cell, and oscilloscope. (c) Waveforms of the applied pulse at a point before (V_a) and after (V_b) the cell, corresponding to the yellow and purple waveforms, respectively. A similar shape of waveforms between these two points confirms that the PCM cell experiences an almost identical pulse duration compared to that of the pulse entering the cell.

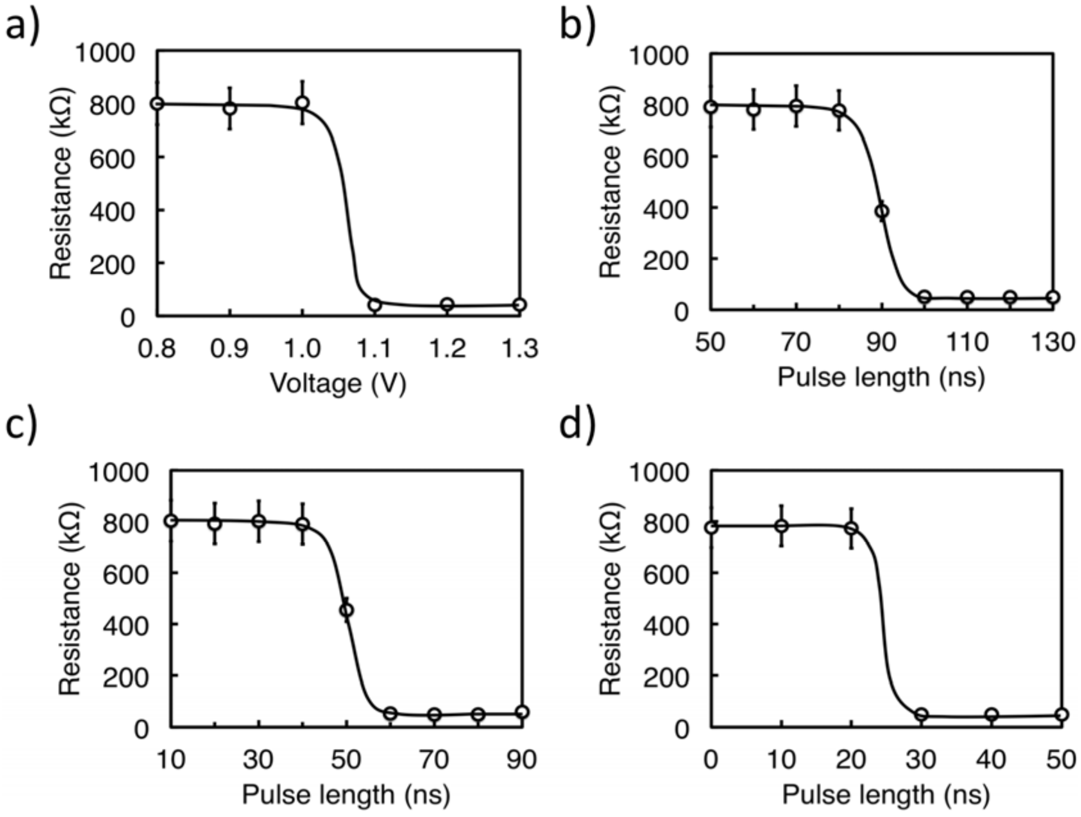

Figure S2. Dependence of the resistance of PCM cells on voltage or pulse length. a) Correlation between resistance and voltage of a test PCM cell, showing V_c ~ 1.0 -1.1 V. The voltage was applied continuously to the cell during the test. b) Resistance vs pulse length for a test cell. The cell was switched from the high-resistance level to the low-resistance level. The voltage was kept constant at 1.1 V. c) Dependence of resistance on the pulse length needed to switch test cells from high- to low-resistance levels. ACT voltage = 1.5 V. d) Correlation between resistance and pulse length of a stimulated cell. The cell was subjected to a stimulus pulse of 1.1V, 10 ns prior to each test. All the error bars show the range of values obtained from 5 repeat experiments performed on a test cell.

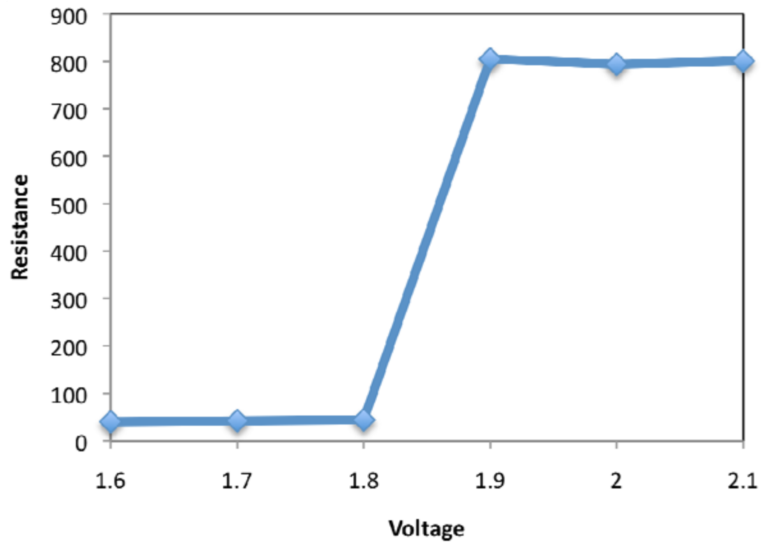

Figure S3. Dependence of a cell resistance on the pulse voltage applied. V_m determined from this figure is 1.8-1.9 V.

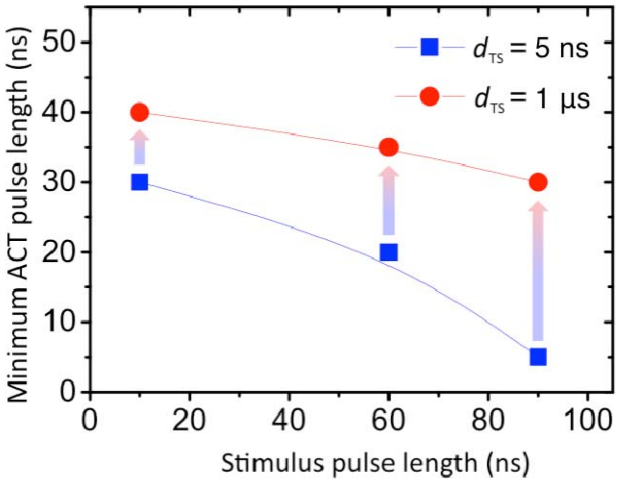

Figure S4. Dependence of the minimum ACT pulse length on the stimulus pulse length. The stimulus and ACT pulse voltages were 1.1 V and 1.5 V, respectively. The degree of relaxation in ACT pulse lengths shows a clear dependence on the stimulus pulse length applied.

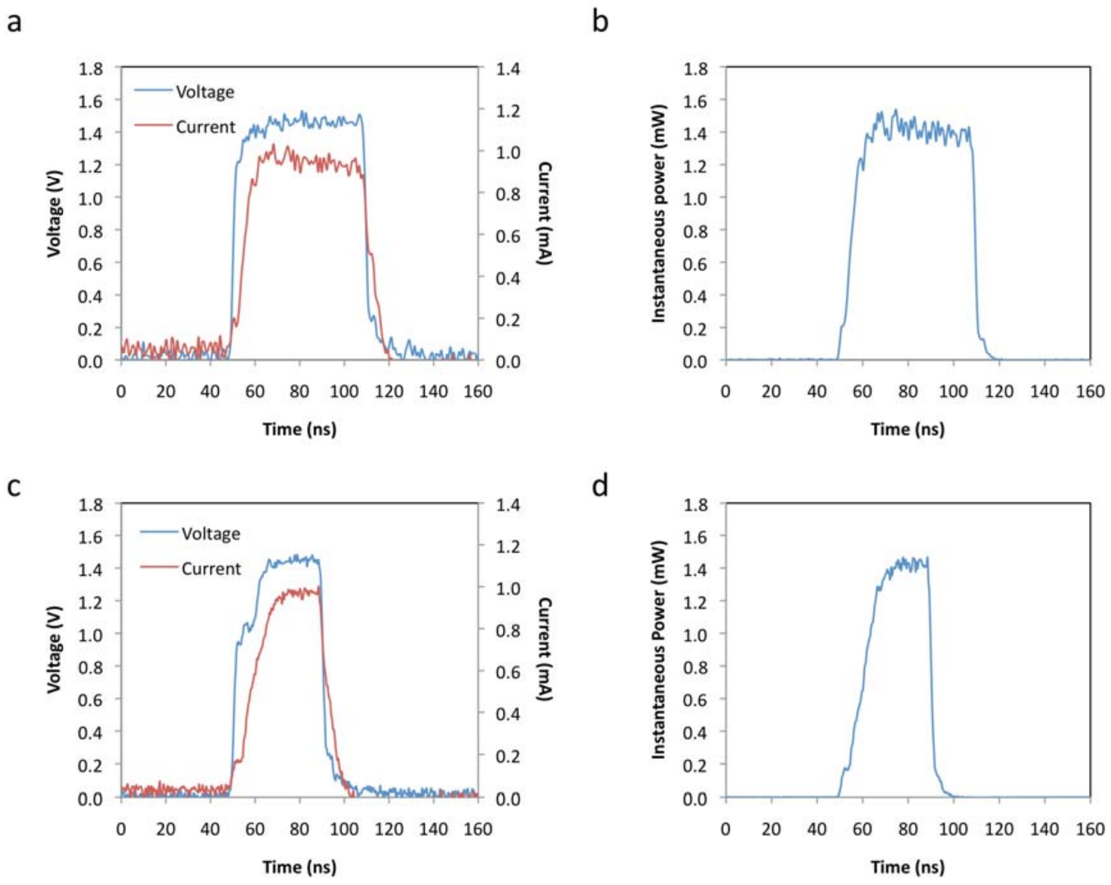

Figure S5. Voltage and current waveforms used to achieve full crystallization for single- and two-pulse set operations. (a) Waveforms and (b) calculated instantaneous power for a single-pulse set operation. The energy consumed was 76 pJ. (c) Waveforms and (d) calculated instantaneous power upon a two-pulse set operation. About 43 pJ of energy was consumed, which corresponds to a 43 % reduction in energy compared to that for a single-pulse excitation.

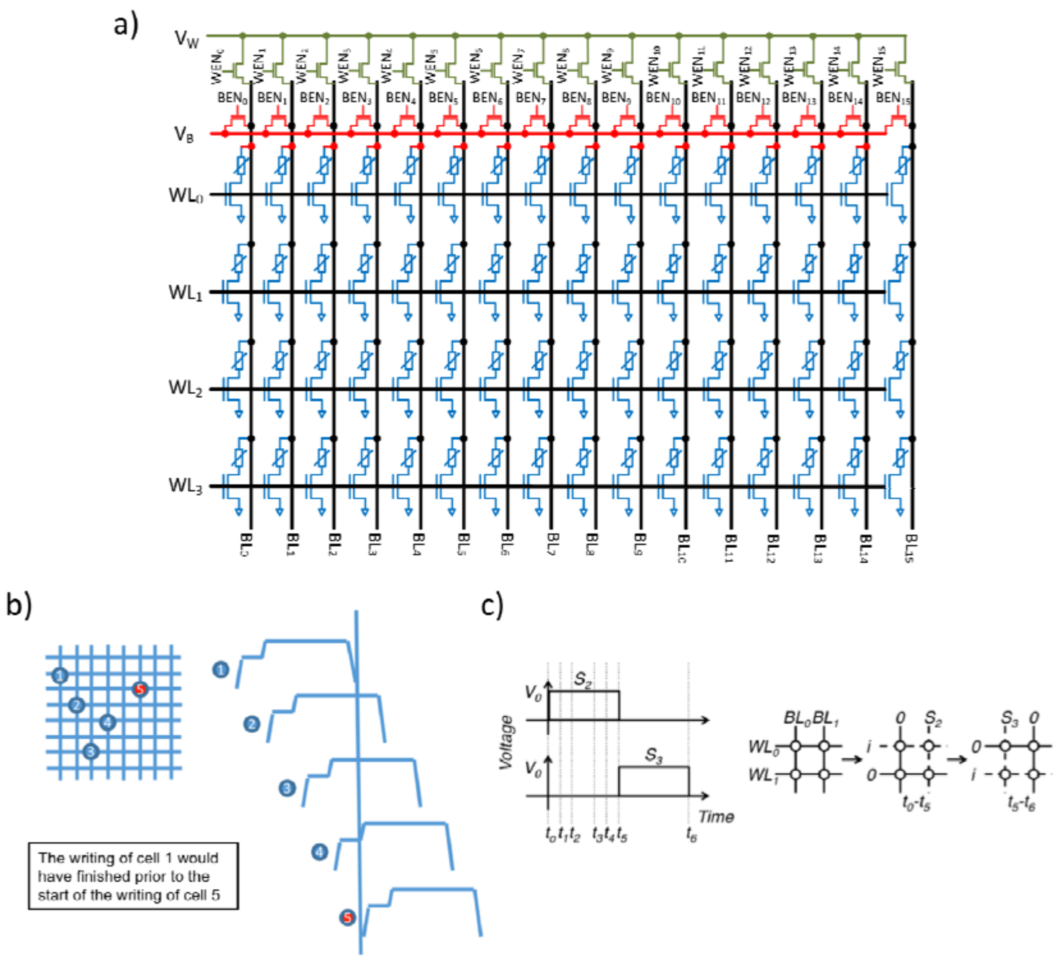

Figure S6. PCM circuits and PL write scheme. (a) Schematic diagram of the stimulating circuit. Note the extra encoder (red) connected to each bit-line for supplying the stimulus pulses (V_B) to the test cells. WL, BL, WEN, and BEN refer to the word line, bit line, write enable, and bias enable, respectively. (b) Waveforms employed in the pipelined (PL) write scheme. (c) Schematic diagram showing the conventional method to switch cells (0,1) and (1,0) in a 2×2 array of test cells. The indicated time steps with different subscripts are given for comparison with the ACT pulse lengths required in our parallel writing scheme, as explained in Figure 2b.

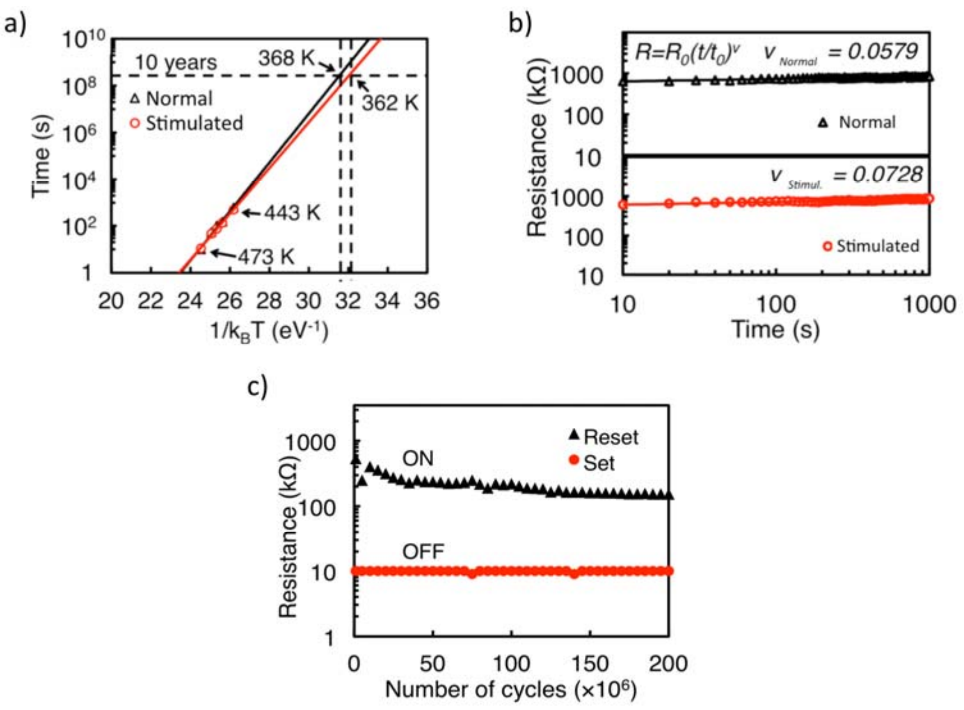

Figure S7. Characteristics of PCM cells. (a) Extrapolated long-term stability of the amorphous phase of an NGST PCM cell. The stimulated cell was subjected to a stimulus pulse of 1.1 V and 60 ns prior to annealing. The cells were annealed at temperatures varying from 443 K to 473 K, and the ACT times were recorded for the normal and stimulated cells. From a data extrapolation, the stimulated cells are estimated to withstand ACT for up to 10 years at 362 K, which is similar to that of normal unbiased cells (i.e. up to 10 years at 368 K). (b) Evolution of PCM cell resistance with time ('drift') in the reset (amorphous) state. The stimulated cell was subjected to a stimulus pulse of 1.1 V and 60 ns prior to the resistance measurements. The upper (lower) panel corresponds to the resistance change of an unstimulated (stimulated) cell as a function of time. (c) Resistance vs number of reset/set endurance cycles for an NGST test cell. The set and reset-pulse lengths were 5 ns. The stimulus time was 1 μs.
